# Mitogenomics, Phylogeny and Morphology Reveal *Ophiocordyceps pingbianensis* Sp. Nov., an Entomopathogenic Fungus from China

**DOI:** 10.3390/life11070686

**Published:** 2021-07-14

**Authors:** Siqi Chen, Yuanbing Wang, Kongfu Zhu, Hong Yu

**Affiliations:** 1 Yunnan Herbal Laboratory, College of Ecology and Environmental Science, Yunnan University, Kunming 650091, China; csq2017@mail.ynu.edu.cn (S.C.); wangyb001@126.com (Y.W.); zkf@mail.ynu.edu.cn (K.Z.); 2 The International Joint Research Center for Sustainable Utilization of Cordyceps Bioresources in China and Southeast Asia, Yunnan University, Kunming 650504, China

**Keywords:** mitochondrial genome, new species, phylogenetic analyses, taxonomy

## Abstract

The new entomopathogenic fungus *Ophiocordyceps pingbianensis*, collected from Southeast China, was described by mitogenomic, morphological, and phylogenetic evidence. The systematic position of *O. pingbianensis* was determined by phylogenetic analyses based on six nuclear gene (ITS, *tef1-α, nrSSU, nrLSU, rpb1* and *rpb2*) and 14 mitochondrial protein-coding gene (PCGs) (*cox*1, *cox*2, *cox*3, *atp*6, *atp*8, *atp*9, *cob*, *nad*1, *nad*2, *nad*3, *nad*4, *nad*5, *nad*6 and *nad*4L) data. Phylogenetic analyses reveal that *O. pingbianensis* was belonged to the *Hirsutella nodulosa* clade in the genus *Ophiocordyceps* of Ophiocordycipiaceae. This fungus exhibits distinctive characteristics which differed from other related *Ophiocordyceps* species with slender and geminate stromata, monophialidic conidiogenous cells with an inflated awl-shaped base, a twisty and warty phialide neck and a fusiform or oval conidia, as well as being found on a tiger beetle of Coleoptera buried in moss at the cave. The complete mitochondrial genome of *O. pingbianensis* was a circular DNA molecule 80,359 bp in length, containing 15 PCGs, 24 open reading frames genes (ORFs), 25 transfer RNA genes (tRNAs) and 27 introns. *Ophiocordyceps pingbianensis*, containing 27 introns, has the second largest mitogenome in Ophiocordycipiaceae and was next to *O. sinensis*. To our knowledge, this is the first report of the mitogenome from a new entomopathogenic fungus, and thus provides an important foundation for future studies on taxonomy, genetics and evolutionary biology of Ophiocordycipiaceae.

## 1. Introduction

*Ophiocordyceps* Petch was belonged to Ophiocordycipitaceae of Hypocreales [1], erected initially by Petch in 1924. In 1931, Petch defined *O. blattae* Petch as a type species [2]. In this genus, the most famous species *O. sinensis* ((Berk.) G.H. Sung, J.M. Sung, Hywel-Jones and Spatafora) is mainly distributed in the high altitudes of the Qinghai-Tibet Plateau [3]. Given the morphological characteristics of asci from several species lacking pronounced apical hemispheric caps and ascospores without being disarticulated into part-spores, *Ophiocordyceps* acts as a subgenus of *Cordyceps* Fr. sensu lato [4,5,6]. Subsequently, the family Ophiocordycipitaceae was established by the type genus *Ophiocordyceps* [1]. Over the last few years, more than 270 species of Ophiocordyceps have been described, making it the largest genus in Ophiocordycipitaceae [1,7,8,9,10,11,12,13]. *Ophiocordyceps* is characterized by a darkly pigmented and tough, wiry, fibrous or pliant stromata ordinarily or obliquely arranged superficial to immersed perithecia; ascospores are commonly cylindrical and multi-septate, disarticulated into part-spores or non-disarticulating [1,14,15].

Asexual morphs of *Ophiocordyceps* consist of *Hirsutella* Pat., *Hymenostilbe* Petch, *Paraisaria* Samson and B.L. Brady, *Sorosporella* Sorokin, *Stilbella* Lindau, as well as *Syngliocladium* Petch [1,14]. The main anamorph of *Ophiocordyceps* refers to *Hirsutella*. The fungal genus Hirsutella was a pathogen of insects, mites and nematodes, which was erected by Patouillard in 1892, and *Hirsutella* was initially described by Petch in 1924. In 1998, Hodge introduced the monograph and molecular phylogeny of *Hirsutella*. To be specific, she reviewed the taxonomy of *Hirsutella* species and described 68 species to pertain to *Hirsutella* [16]. *Hirsutella* was considered to show relationships to the genus *Ophiocordyceps* typified by a sexual morph [1]. The morphological characteristics of this genus was considered to produce basally inflated phialides, tapering towards the apex, discontinuous hymenial layer and conidia embedded in a mucous sheath [15,17,18].

The mitochondrion acts as a master regulator of metabolism. As DNA sequencing technology leaps forward, the mitochondrial DNA (mtDNA) is recognized as an effective marker for phylogenetic analysis, with the advantage of exhibiting a faster mutation rate than nuclear DNA [19]. Moreover, mtDNA is also appropriate for phylogenetic analysis due to its high copy number and conservative gene functions [20].

Furthermore, there are differences in the size of fungal mitochondria in different species. From the current reports, the smallest mitogenome known in the fungi was 12.1 kb in *Rozella allomycetis* (Doweld) Letcher in Cryptomycota [21], while the largest was 272,238 bp in *Morchella importuna* M. Kuo, O’Donnell and T.J. Volk in Pezizomycetes [22]. The mitochondrial genome of the Ophiocordycipitaceae species is like an obvious closed circular structure, comprising naked double stranded DNA. It commonly contains 15 protein-coding genes (e.g., 3 subunits of cytochrome c oxidase (cox1–3), 3 subunits of ATP synthase (*atp*6, 8 and 9), cytochrome b gene (*cob*), 7 subunits of NADH dehydrogenase (*nad*1–6, *nad*4L) and 1 ribosomal protein S3 (*rps*3), as well as ribosomal RNA genes (*rnl, rns*) and transfer RNA genes (*trn*). Thus far, mitogenomes of only ten species in Ophiocordycipitaceae have been described, in which mitogenomes of *Hirsutella* species have been rarely reported. The existing sequence data of mitogenome from NCBI only include *H. minnesotensis* Sen Y. Chen, Xing Z. Liu ad F.J. Chen, *H. rhossiliensis* Minter and B.L. Brady, *H. thompsonii* F.E. Fisher and *H. vermicola* M.C. Xiang and Xing Z. Liu. The largest mitogenome known reached 157.5 kb in *O. sinensis* [23], and the smallest was 52,245 bp in *H. minnesotensis* [24]. Different variations of mitogenome sizes of hypocrealean fungi were largely attributed to differences in the number/length of introns and the length of intergenic regions, and the mentioned introns most likely were obtained through horizontal transfer from other fungal species [25].

In the present study, an unknown species of *Ophiocordyeps* attacking a larva of tiger beetle was collected from Daweishan National Nature Reserve, Pingbian County, Yunnan Province, China. Mitogenomics, phylogeny and morphology of this fungus were determined, and its systematic position was established in Ophiocordycipitaceae, It was revealed a new species of *Ophiocordyceps* with a *Hirsutella* morph. The complete mitogenome of the new fungus was sequenced, assembled and annotated. Genome structures, gene contents, codon usage and gene arrangement were analyzed.

## 2. Materials and Methods

### 2.1. Fungal Materials and Isolation

In this study, live specimens were collected on a tiger beetle of Coleoptera buried in the moss in a cave in Daweishan National Nature Reserve, Pingbian County, Yunnan Province, China. Before examination, the specimen was transferred to a laboratory and then stored at 4 °C. To detect and further study the culture, stromata were photographed and then measured with an Olympus SZ61 stereomicroscope. A stroma of the fungus growing from the beetle larva fell to small segments nearly 5 mm long. The respective segment was surface-sterilized in 30% H_2_O_2_ for 5 min, then soaked in 70% alcohol for 2 min and subsequently rinsed sufficiently in sterilized water, and lastly dried on the sterilized filter paper. Afterwards, the segments were placed on Potato Dextrose Agar (PDA) plates. The specimen was deposited in Yunnan Herbal Herbarium (YHH), Yunnan University. The cultures of this fungus were deposited in Yunnan Fungal Culture Collection (YFCC), Yunnan University.

### 2.2. Morphological Observations

The fresh specimen, including stromata and host, were photographed with Olympus CX40 and BX53 microscopes. The colonies on PDA plates were cultured at 4 °C for 4 weeks, and the colonies characteristics (size, texture and color) were photographed with the Cannon 700D camera to characterize the morphology of colonies. For to asexual morphological descriptions, microscope slide cultures were prepared by placing a small amount of mycelia on 5-mm diameter PDA medium blocks that were overlaid by a cover slip. The cultures on the slants were transferred to PDA plates and then cultured with an incubator for 25 d at 4 °C. Next, the colonies were photographed and then measured every four days. The micro-morphological observations and measurements were performed under Olympus CX40 and BX53 microscopes, as well as a FEI QUANTA200 scanning electron microscope [26].

### 2.3. DNA Extraction, PCR and Sequencing of Nuclear Genes

Axenic living cultures of the new species were collected from a PDA plate to prepare for DNA extraction. The DNA extraction was performed with the CTAB method in Liu et al. [27] The nuclear ribosomal small subunit (*nrSSU*) was amplified with the primer pair nrSSU-CoF and nrSSU-CoR [28]. The nuclear ribosomal large subunit (*nrLSU*) and translation elongation factor 1α (*tef-1α*) were amplified with the primers LR5 and LR0R [29,30] and EF1α-EF and EF1α-ER [1,30], respectively. To amplify the largest subunits of RNA polymerase ІІ (*rpb1*) (*rpb2*), the primer pair RPB1–5′F and RPB1–5′R, as well as the primer pair RPB2–5′F and RPB2–5′R, were applied respectively [31]. The nuclear ribosomal internal transcribed spacer region (ITS) was amplified using the primer pair ITS4 and ITS5 [32]. The polymerase chain reaction (PCR) assay was performed as described by Wang et al. [33]. PCR products were sequenced with an automatic sequence analyzer (BGI). When PCR products could not be sequenced directly, cloning was performed with the TaKaRa PMD™18-T vector system (TaKaRa Biotechnology, Dalian, China).

### 2.4. Sequencing, Assembly and Annotation of Mitogenome

After the DNA extraction and the quality detection, the extracted genomic DNA was transported on dry ice for high-throughput sequencing at Novogene Co., Ltd. (Beijing, China) by applying dry ice for high-throughput sequencing. Following standard procedures, the extracted genomic DNA was sequenced on the Illumina HiSeq 3000 platform using a 350 bp paired-end reads, which resulted in 3 GB of data and 100× sequencing depth. Using the high-throughput sequencing data, mitogenome of the new species was assembled with the software SPAdes v. 3.11.0 [34], which involved three processes, i.e., (1) initial splicing, (2) contig screening and extension and (3) hole filling and re-splicing. The complete mitogenome of the new species was annotated by combining the results of a MFannot tool [35] and an ARWEN web server, with artificial correction. The content of the annotated sample mainly included the protein coding genes (PCGs), the rRNA genes, the tRNA genes and the open reading frames (ORFs). Next, the graphical map of the complete mitogenome of the new species was generated with the software Organellar Genome DRAW tool [36].

### 2.5. Sequence Analyses of Mitogenome

The GC content of the mitochondrion sequence was analyzed using Mega 7. tRNA genes were analyzed with tRNAscan-SE 2.0 (http://lowelab.ucsc.edu/tRNAscan-SE/index.html, accessed on 1 October 2020). Sequence Manipulation Suite (http://www.detaibio.com/sms2/codon_usage.html, accessed on 20 October 2020) and CodonW 1.4.4 were adopted to calculate codon usage in the mitogenome. Genomic synteny analysis of six Ophiocoryceps species was conducted using Mauve v2.4.0 (A.C. Darling, 2004).

### 2.6. Phylogenetic Analyses

Raw trace files were inspected and edited using MEGA7. Two species of *Tolypocladium inflatum* W. Gams and *T. ophioglossoides* J.F. Gmel were designated as the outgroup to root the trees. Five gene (*nrSSU, nrLSU, tef-1α, rpb1* and *rpb2*) sequences of *Hirsutella*, *Ophiocordyceps* and the outgroup, were retrieved from GenBank. The majority of ITS sequences of *Hirsutella* were also retrieved from GenBank. Some manual adjustments were made as the nrSSU gene contained an intron (Table S1). The sequences were aligned with the programmes Clustal X2.0 and MEGA7 [37,38]. Phylogenetic analyses were performed by combining the concatenated 14 PCGs sequences generated here with other mitogenomic sequences of species in Hypocreales downloaded from NCBI. *Neurospora crassa* Shear and B.O. Dodge and *Podospora anserine* Rabenh were designated as the outgroup. The 14 mitochondrial PCGs were aligned using MUSCLE. [39]. Maximum likelihood (ML) and Bayesian Inference (BI) phylogenetic trees were estimated using RaxML 7.0.3 and MrBayes v.3.1.2. [26,40,41].

## 3. Results

### 3.1. Phylogenetic Analyses Based on Nuclear Genes

**Figure 1:**
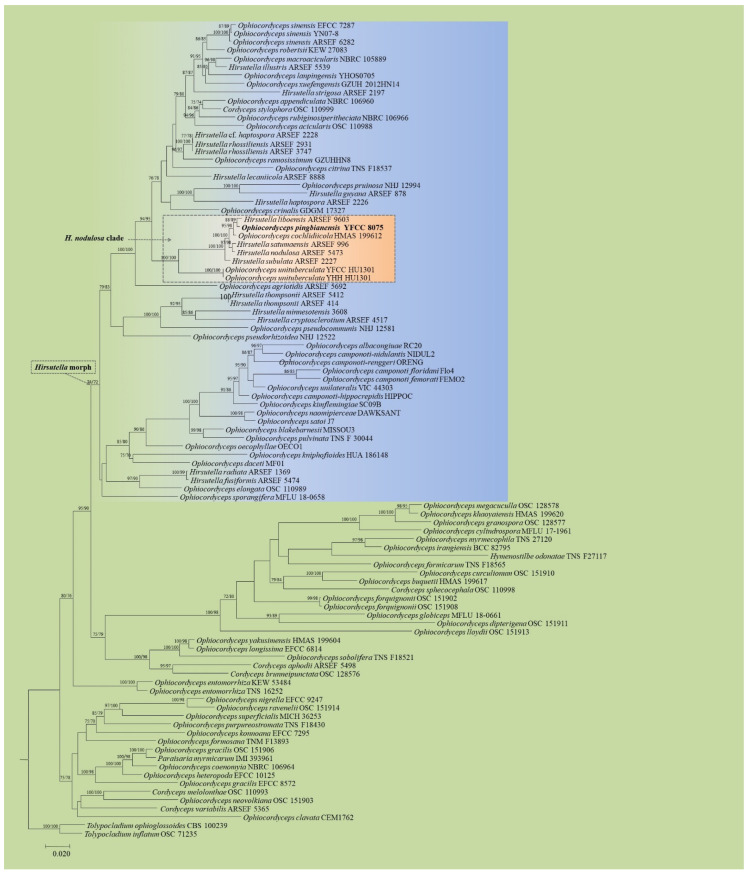
Phylogenetic tree of *Ophiocordyceps* inferred from Maximum Likelihood (ML) and Bayesian Inference (BI) analyses based on a five-gene (*nrSSU, nrLSU, tef-1α, rpb1* and *rpb2*) dataset. Values at the nodes before and after the backslash are ML bootstrap proportions and BI posterior probabilities, respectively. Support values greater than 50% are indicated at the nodes. Phylogenetic tree shows the placement of *O. pingbianensis* within the genus *Ophiocordyceps. Tolypocladium inflatum* and *T. ophioglossoides* are designed as the outgroup.

**Figure 2:**
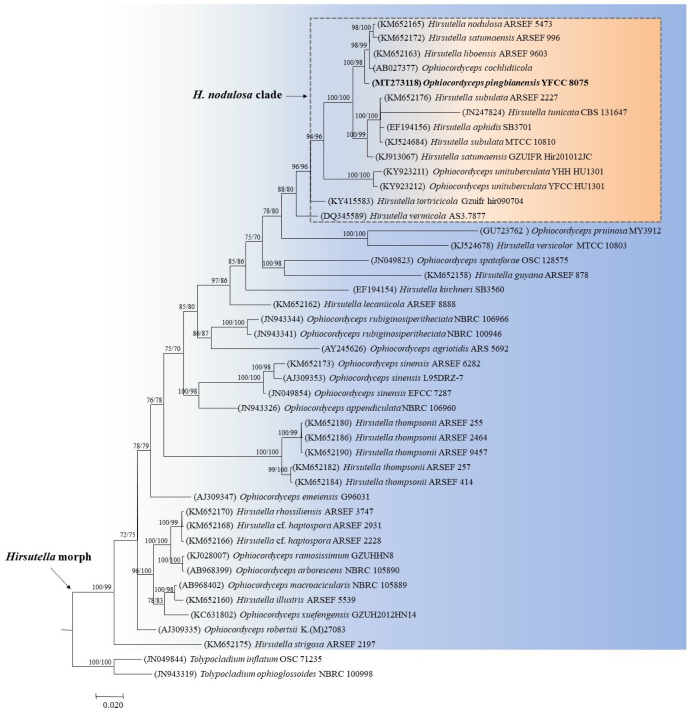
Phylogenetic relationships of *Ophiocordyceps pingbianensis* to the related taxa from ML and BI analyses based on ITS sequences. Values at the nodes before and after the backslash are ML bootstrap proportions and BI posterior probabilities, respectively. Support values greater than 50% are indicated at the nodes. Phylogenetic tree shows that *O. pingbianensis* belongs to the *H. nodulosa* clade in the genus *Ophiocordyceps* with *Hirsutella* morph. *Tolypocladium inflatum* and *T. ophioglossoides* were designed as the outgroup. GenBank accession numbers for ITS sequences are placed in the bracket before the taxon name.

For the ML and BI trees estimated for the five nuclear genes, the new species was closely related to *H. liboensis* X. Zou, A.Y. Liu and Z.Q. Liang and *O. cochlidiicola* (Kobayasi) G.H. Sung, J.M. Sung, Hywel-Jones and Spatafora, clustered as a separate clade from other allied species in the genus *Ophiocordyceps* (Figure 1). For the ML and BI trees estimated for the ITS sequences, there was no significant different in topology between the five-gene and ITS phylogenetic trees. The new species formed an independent clade from its allied species of *Hirsutella* and was closely grouped with *H. liboensis* and *O. cochlidiicola* (Figure 2).

### 3.2. Phylogenetic Analysis Based on Mitochondrial Genes

**Figure 3:**
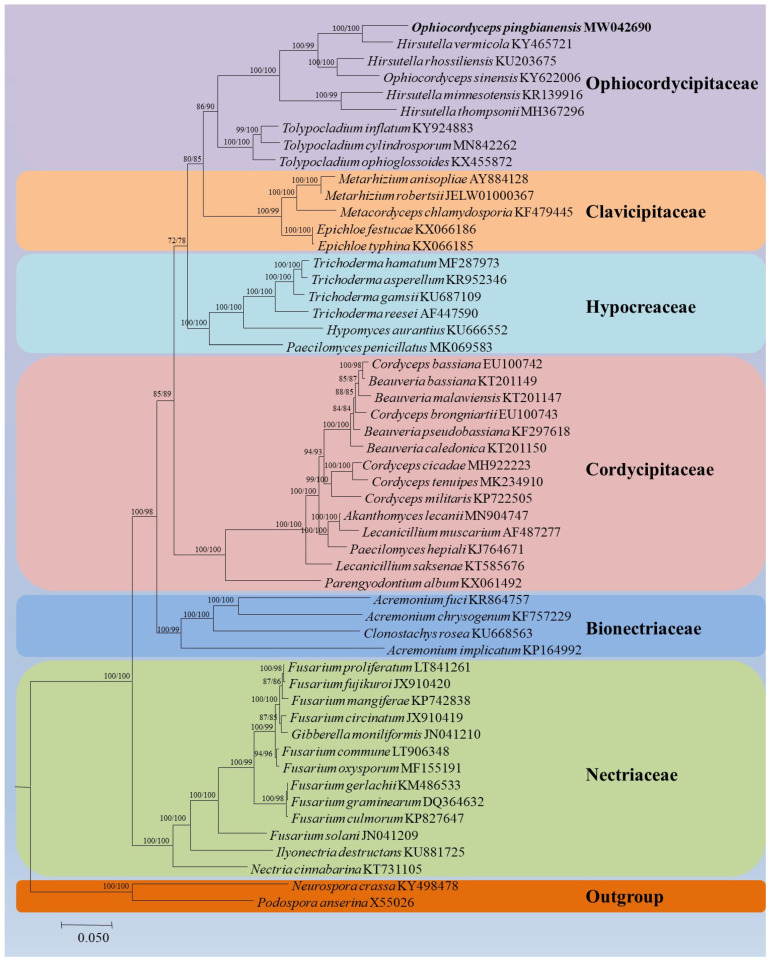
Phylogenetic relationships among 53 taxa of Sordariomycetes based on ML and BI analyses from 14 concatenated mitochondrial protein-coding genes (PCGs). The 14 PCGs included subunits of the respiratory chain complexes (*cob, cox1, cox2, cox3*), ATPase subunits *(atp6, atp8, atp9*), NADH: quinone reductase subunits (*nad1, nad2, nad3, nad4, nad4L, nad5, nad6*). Values at the nodes before and after the backslash are ML bootstrap proportions and BI posterior probabilities, respectively. Support values greater than 50% are indicated at the nodes. Phylogenetic tree shows the placement of *O. pingbianensis* in the family Ophiocordycipitaceae. *Neurospora crassa* and *Podospora anserina* were designed as the outgroup.

The ML tree and BI tree were estimated for the mitochondrial PCG dataset of 52 species in Ascomycota from GenBank (Table S2). The six well-supported clades of Hypocreales were recognized by ML and BI analyses, including species of the families Bionectriaceae, Clavicipitaceae, Cordycipitaceae, Hypocreaceae, Nectriaceae and Ophiocordycipitaceae (Figure 3). As indicated from the phylogenetic analysis, the new species was a member of the family Ophiocordycipitaceae. The new species was clustered together with *H. minnesotensis* and *H. vermicola* in the genus *Ophiocordyceps* of Ophiocordycipitaceae.

### 3.3. Taxonomy

**Figure 4:**
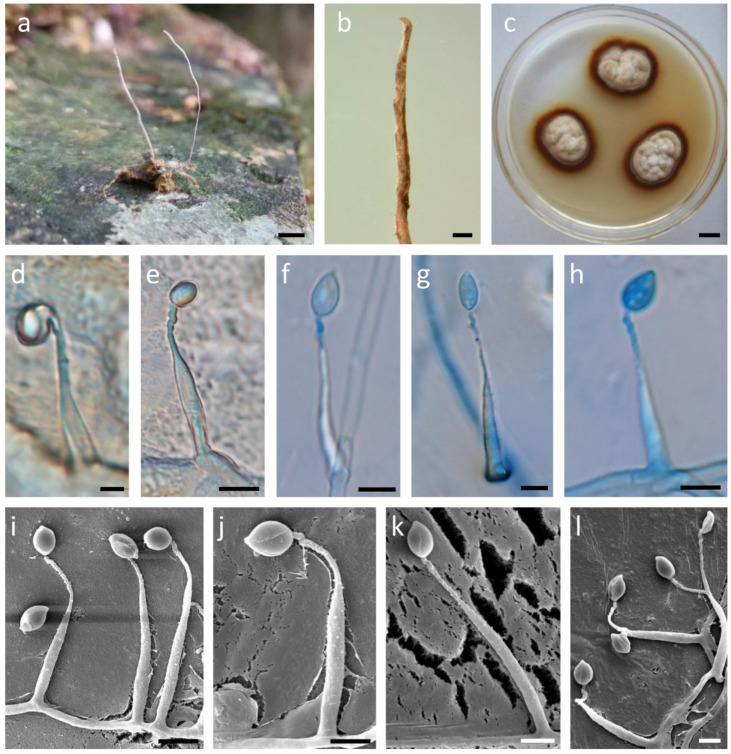
Ecology and morphology of *Ophiocordyceps pingbianensis*. (**a**) Stromata arising from a Larva of tiger beetle of Coleoptera; (**b**) The tip of a stroma; (**c**) Colony on PDA medium; (**d**–**l**) Conidiogenous cells. Scale bars: (**a**) = 0.5 cm; (**b**) = 500 µm; (**c**) = 1 cm; (**d**–**l**) = 5 µm.

***Ophiocordyceps pingbianensis*** H. Yu, S.Q. Chen and Y.B. Wang, sp. nov. (Figure 4)

**MycoBank:** MB 840055.

**Etymology:** Named after the location Pingbian County where this species was collected.

**Holotype:** China. Yunnan Province: Pingbian County, Daweishan National Nature Reserve, at 103°41ʹ57″ E, 22°57ʹ45″ N, alt. 1536 m, on a tiger beetle of Coleoptera buried in the moss in a cave, 17 June 2019, Y.B. Wang (YHH 18766, holotype; YFCC 8075, ex-holotype living culture).

Stromata geminate, slender, smooth and unbranched, flexible to ligneous, with surface color from light deep yellow to dark brown, 17–21 (x = 19) mm in length, 0.16–0.21 (x = 0.19) mm in width. Colonies on PDA slow-growing, with a diam up to 18–20 mm after 4 weeks at 4 °C, lanate, consisting of a loose mycelial felt with annulation, beige to pale brown, dark brown at the edge. After 90 d of culture, stromata were not observed. Hyphae hyaline, septate, branched, smooth-walled, 2.8–4.4 μm (x = 3.6) in width. Conidiogenous cells monophialidic, non-septate, arising from hyphae laterally, with an inflated awl-shaped base, 20.4–31.6 μm (x = 26.0) long, 3.2 –5.2 μm (x = 4.2) wide at the base, phialide neck twisty and warty, 0.6–1.4 μm (x = 1.0) wide at the apex. Conidia solitary, hyaline, aseptate, smooth-walled, fusiform or oval, 3.1–5.3 × 4.6–7.5 μm (x = 3.9 × 6.4), embedded in a mucous sheath of 6.5–8.6 × 7.1–9.2 μm (x = 6.8 × 8.9).

**Host:** Larva of a tiger beetle (Coleoptera).

**Habitat:** Buried in the moss in a cave.

**Type locality:** Daweishan National Nature Reserve, Pingbian County, Yunnan Province, China.

**Sexual stage:** The fruiting body was not observed.

**Notes:***Ophiocordyceps pingbianensis* is characterized by geminate stromata, slender, unbranched and a surface color from light deep yellow to dark brown; monophialidic conidiogenous cells with an inflated awl-shaped base, twisty and warty phialide neck, fusiform or oval conidia. Morphological comparison showed that *O. pingbianensis* was distinct from other closely related species (Table 1), which was concordant with molecular phylogenetic results.

### 3.4. Mitogenomic Characteristics of Ophiocordyceps Pingbianensis

**Figure 5:**
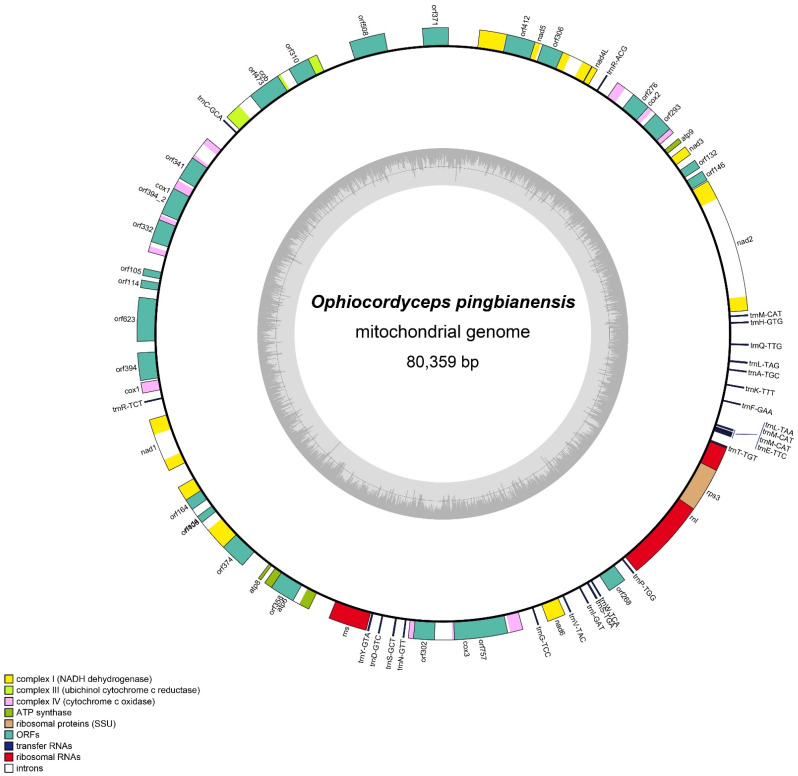
The mitogenomic circular map of the complete mitochondrial genome of *Ophiocordyceps pingbianensis*. Genes are represented with different color blocks.

The complete mitogenome of *O. pingbianensis* was an obvious circular molecule with a length of 80,359 bp (Figure 5). It had an overall GC content of 29.89%. The mitogenome of *O. pingbianensis* was a rather high capacity and compacted genome with genic regions (including intronic regions) accounting for 71.54% and intergenic regions taking up 27%. The protein-coding regions took up 92.19% of the whole mitogenome of *O. pingbianensis,* containing 2 rRNA genes (i.e., *rnl* and *rns*) genes, 15 standard protein-coding genes (PCGs), 24 ORFs, 25 transfer genes (tRNAs) (Table S3). The sizes of *rns* and *rnl* were 1652 bp and 6444 bp, respectively. The total length of the 15 PCGs was 14682 bp, including 3 subunits of cytochrome c oxidase (*cox*1, *cox*2 and *cox*3), 3 subunits of ATP synthase (*atp*6, *atp*8 and *atp*9), one cytochrome b gene (*cob*) and seven subunits of NADH dehydrogenase complex (*nad*1, *nad*2, *nad*3, *nad*4, *nad*5, *nad*6 and *nad*4L) and 1 ribosomal protein S3 (*rps*3). The lengths of 25 tRNAs ranged from 69 to 84 bp and 24 ORFs (*orf*146, *orf*132, *orf*293, *orf*276, *orf*306, *orf*412, *orf*371, *orf*508, *orf*310, *orf*473, *orf*341, *orf*394_2, *orf*332, *orf*105, *orf*114, *orf*623, *orf*394, *orf*164, *orf*108, *orf*374, *or*f358, *orf*302, *orf*757 and *orf*268).

### 3.5. Codon Usage

The codon usage of mitochondrial genes (*atp*6–9, *cob*, *cox*1–3, *nad*1–6, *nad*4L, *orf*146, *orf*132, *orf*293, *orf*276, *orf*306, *orf*412, *orf*371, *orf*508, *orf*310, *orf*473, *orf*341, *orf*3942, *orf*332, *orf*105, *orf*114, *orf*623, *orf*394, *orf*164, *orf*108, *orf*374, *or*f358, *orf*302, *orf*757 and *orf*268) of *O. pingbianensis* was determined. The frequency of initiation codon ATG was the highest, followed by AAA, *orf*473, *orf*394_2, *orf*332 and *orf*302 started with “AAA”, *orf*146, *orf*293 and *orf*341 started with “AGA”, *orf*164 and *orf*358 started with “AAC”. The frequency of stop codon TAA was the highest, followed by TAG. Only *cox1* was terminated by “GCT” (Table S4). Leucine was the most common amino acid in the PCGs in the mitogenome of *O. pingbianensis*, followed by Arginine (Table S5). For codon usage, the most commonly used codons were UUA (3.88%), AGA (3.6%), CCU (2.37%), GCU (1.99%), UCU (1.94%), and GGU (1.93%), and the least used codons included CUG (0.19%), AUC (0.18%), ACC (0.17%), CGG (0.15%) and CUC (0.11%).

### 3.6. Transfer RNAs

**Figure 6:**
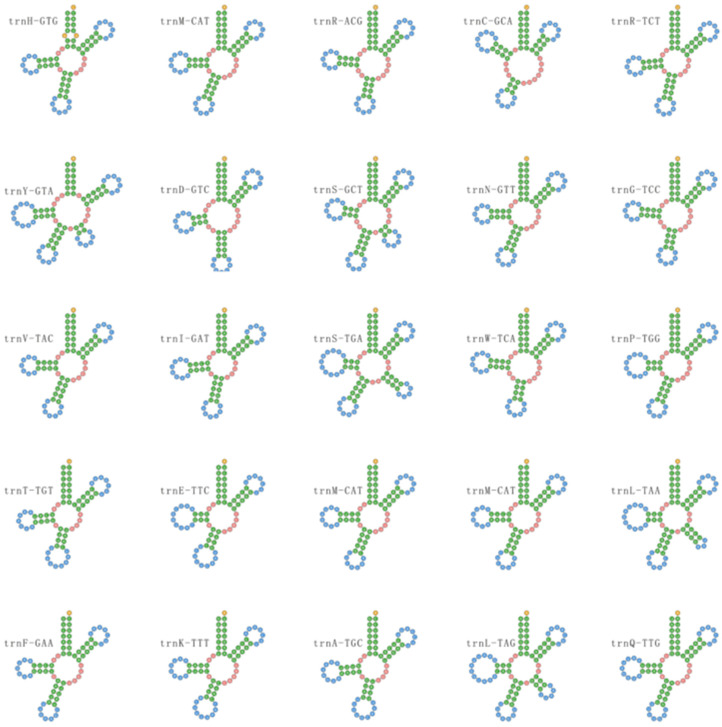
The predicted tRNA structures of *Ophiocordyceps pingbianensis*. Secondary structures predicted by tRNA scan-SE show that all tRNAs adopt a typical cloverleaf structure except for *trn*S-GCT, *trn*Y-GTA, *trn*L-TAA, *trn*L-TAG and *trn*S-TGA.

In the mitogenome of *O. pingbianensis*, the 25 tRNAs coded for all 20 amino acids (Table S6). As indicated from the results, all tRNAs adopt a typical cloverleaf structure except for *trn*S-GCT, *trn*Y-GTA, *trn*L-TAA, *trn*L-TAG and *trn*S-TGA (Figure 6). As shown in Table S6, some of the 25 tRNAs existed in multiple copies, the *trn*M-CAT gene for methionine appeared in three copies. Two different tRNAs for arginine (*trn*R-ACG and *trn*R-TCT), Serine (*trn*S-GCT and *trn*S-TGA) and Leucine (*trn*L-TAA and *trn*L-TAG) were found.

### 3.7. Gene Arrangement Analysis

**Figure 7:**
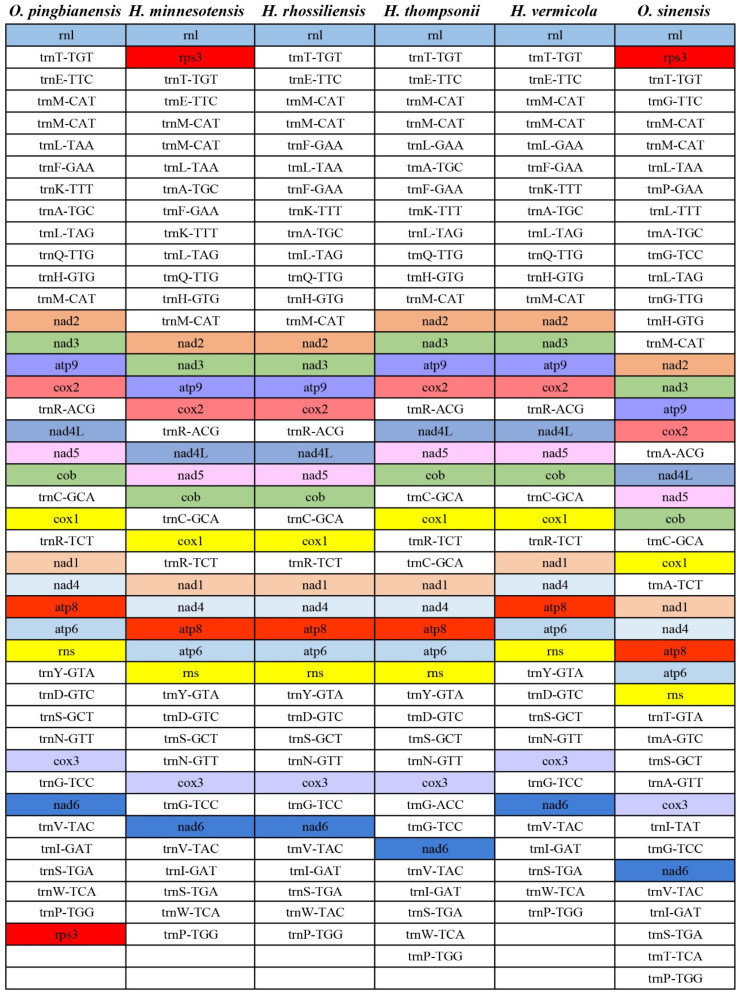
A comparison of gene order among six *Ophiocordyceps* mitogenomes. The genes are colored on the basis of their functional groups. The non-coding region (NCR) is not indicated.

**Figure 8:**
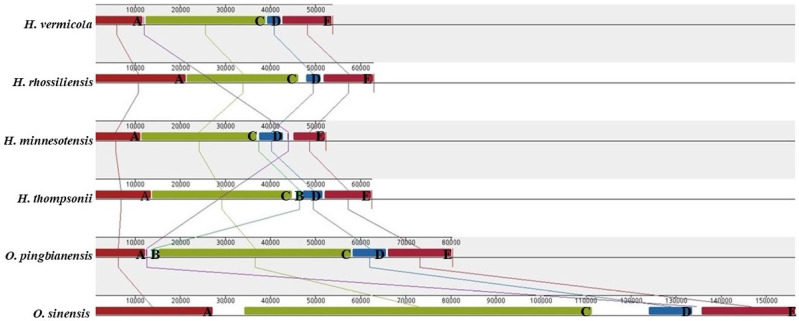
Mitogenome collinearity analysis of six *Ophiocordyceps* species. The progressive Mauve alignment shows the homologous regions shared among the mitogenomes, and it also connected these regions with lines, indicating their corresponding positions among the homologous regions in order to visualize the gene arrangement. (**A**–**E**) represent different homologous regions.

Overall, the lative positions of most PCGs and tRNAs were relatively conservative across the six *Ophiocordyceps* species (Figure 7). However, several tRNAs and PCGs were distributed in different positions in six mitogenomes, showing the variability of the relative gene order of these mitogenomes. As revealed from the results of genomic synteny (Figure 8), the six *Ophiocordyceps* mitogenomes fell to 4–5 homologous regions. Homologous regions were identified in the mitogenome of *O. pingbianensis* and *H. minnesotensis,* adding the homologous region B. However, there was an absence of the homologous region B in mitogenomes of *O. sinensis*, *H. thompsonii*, *H. vermicola* and *H. rhossiliensis*. The relevant orders of four homologous regions A, C, D and E were conserved across the six *Ophiocordyceps* mitogenomes. The homologous regions B was distributed in different positions of all the mitogenomes.

## 4. Discussion

By morphological and molecular phylogenetic researches, *O. pingbianensis* was found as a novel species, being belonged to the *H. nodulosa* clade. Morphological differences still exist between *O. pingbianensis* and its related species, such as the conidiogenous cells of *H. liboensis* with being smooth and polyphialidic [43]. *O. pingbianensis* has a warty phialide neck of conidiogenous cells, whereas a larger periclinal protuberance was produced near the apex of conidiogenous cells of *O. unituberculata* H. Yu, Y.B. Wang and Y.D. Dai [18]. Conidia of *O. pingbianensis* were smooth, fusiform or oval, inconsistent with *O. unituberculata* with larger lanceolate to fusiform conidia and *H. nodulosa* with non-mucoid conidia. The host of *O. pingbianensis* was a tiger beetle of Coleoptera; however, these hosts of its closely related species *H. satumaensis, H. nodulosa, H. liboensis* and *O. cochlidiicola* are Lepidoptera.

The mitogenome of *O. pingbianensis* was a circular DNA molecule with a length of 80,359 bp, and the mitogenome size was the second largest within the mitogenomes of the Ophiocordycipitaceae species reported. The mitogenome sizes were different among species in *Ophiocordyceps* attributed to differences in the number of introns. For instance, the *H. thompsonii* mitogenome exhibited 15 introns [25], the *H. rhossiliensis* mitogenome had 13 introns [48], the *H. minnesotensis* mitogenome had 13 introns [24] and the *H. vermicola* mitogenome exhibited 7 introns [47]. The mitogenome size of *O. pingbianensis* was the second largest because it contained 27 introns. The mitochondrial genome of H. minnesotensis exhibited significant intron degeneration for the nad4L and cox1-i1 genes, which might be caused by unexpected stop codons or frame shifting [24]. In *H. rhossiliensis*, there were 10 group I introns and one unclassified intron in six genes (i.e., *rnl*, *cob*, *cox*1, *cox*3, *nad*1 and *nad*5) [48]. This indicated an intron presence/absence dynamics cause mitogenome size variations in the *Ophiocordyceps* species. On the whole, 27 ORFs were identified in *O. pingbianensis*, and except for *orf*144, *orf*131, all ORFs were transcribed at the identical orientation. In the mitogenomes of five *Ophiocordyceps* species reported, the number of ORFs was also different, e.g., the *H. thompsonii* mitogenome containing 3 ORFs [25], *H. rhossiliensis* mitogenome containing 5 ORFs [48], *H. minnesotensis* mitogenome containing 4 ORFs [24], as well as the *H. vermicola* mitogenome containing 3 ORFs [47].

To gain more insights into the variability of the mitochondrial gene among *Ophiocordyceps* species, the genomic arrangements of *O. pingbianensis* with other five mitogenomes in *Ophiocordyceps* were analyzed comparatively. The lative positions of most PCGs and most tRNAs of the six *Ophiocordyceps* species were relatively conservative, except for several tRNAs and PCGs distributed in different positions, which resulted from the variability of the relative gene order of these mitogenomes.

As revealed from genomic synteny, the six *Ophiocordycep* mitogenomes falled to 4–5 homologous regions. Homologous region B only existed in the mitogenome of *O. pingbianensis* and *H. minnesotensis,* however, it was lacked in *H. thompsonii*, *H. vermicola, H. rhossiliensis* and *O. sinensis* mitogenomes. The relevant orders of four homologous regions (i.e., A, C, D and E)were conserved across the six *Ophiocordyceps* mitogenomes and homologous regions B were distributed in different positions among the mitogenomes; as a result, the diversity of gene order was generated among six mitogenomes. Gaining insights into gene arrangements, component and genomic synteny of *Ophiocordyceps* species might help assemble mitogenomes and trace the evolutionary history of other *Ophiocordycipitaceae* species in future research.

## Figures and Tables

**Table 1:** A morphological comparison of *Ophiocordyceps pingbianensis* and its related species.

Species	Host	Habitat	Synnemata/Stromata	Conidiogenous Cells	Conidia	References
*H. aphidis*	Aphids	On plant	Small or as short cushions	Narrowly conical, length (17–90 µm), neck width (0.6–1.0 µm), base width (2.6–4.0 µm)	Slightly crescent-shaped or cymbiform, usually in pairs, 7.0–18.3 × 2.8–4.6 µm, with mucous sheath	[42]
*H. liboensis*	Larvae of Cossidae (Lepidoptera)	In tree hole	Clustered	Base significantly swollen, length (28–30 µm), neck width (1–2 µm), twisting in 2–3 helices at the apex, base width (3–4.5 µm)	Fusiform or orange-segmented, in pairs or single, 6–8 × 3–5 µm, with mucous sheath	[43]
*H. nodulosa*	Lepidoptera, Acari	On plant	None	Base swollen, length (20–35 µm), base width (4 µm), neck often twists in a helix at the apex, neck width (1 µm), with tiny warts	Ellipsoid or like orange segments, 5–6 × 3 µm, with mucous sheath	[44]
*O. pingbianensis*	Larvae of tiger beetle (Coleoptera)	Clinging to fallen leaves	Geminate, 17–21 × 0.16–0.21	Base obviously swollen, length (20.4–31.6 μm), base width (3.2–5.2 μm), neck width (1–1.5 µm), twisty and warty at the apex	Solitary, fusiform or oval, 5.3–7.5 µm long, 3.14.6 µm wide, with mucous sheath	This study
*H. satumaensis*	Larvae of *Bombyx mori* (Lepidoptera)	On plant	Clustered,3.0–6.5 × 0.5 mm.	With conoid to cylindrical base, size (5–17 × 3–4.5 μm), neck length (7 µm), twisting in a helix at the apex; base with warts	Fusiform or like orange segments, 5–7.5 × 3–5μm, with mucous sheath	[14,45]
*H. subuluta*	Larvae of Lepidoptera	On plant	Solitary, 15–50 × 0.1–0.3 mm	Phialidic, neck 6–12 µm long, base size (4–8 × 3–5 µm)	Narrowly ellipsoid, in pairs or single, 4–8 × 1.5–2.5 μm, with mucous sheath.	[16]
*H. tortricicola*	Larvae of Tortricidae (Lepidoptera)	In wilted leaf	Solitary	Base obviously swollen, length (18–22 µm), base width (3.5–4 µm), neck width (1–1.5 µm), neck often twists in 1–2 helices at the apex	Ellipsoid or like orange segments, in pairs or single, 2.7–3.6 × 1.4–1.8 μm, with mucous sheath	[46]
*O. unituberculata*	Larvae of Noctuidae (Lepidoptera)	In soil or cling to the fallen leaves	Clustered, 5–76 × 0.4–0.7 mm	With an inflated awl-shaped base, length (31.9–128.3 µm), base width (1.8–5.0 µm), neck width (0.5–1.2 µm). With a large periclinal protuberance near the apex	Solitary, lanceolate to fusiform, 6.3–10.6 × 1.9–3.7 µm, with mucous sheath	[18]
*H. vermicola*	Bacteria-feeding nematodes	In soil	None	Base obviously swollen, length (14–26 µm), base width (3.0–5.0 µm), neck width (1–2 µm), neck often twists in a helix towards the apex	More or less ellipsoid, single or in groups of 2–8 × 3–5 µm, with mucous sheath	[47]

## Data Availability

Not applicable.

## Supplementary Materials

The following are available online at https://www.mdpi.com/article/10.3390/life11070686/s1, Table S1: Specimen information and GenBank accession numbers for sequences used in this study. Table S2: Specimen information and GenBank accession numbers for mitogenomes used in this study. Table S3: General features in the mitogenome of Ophiocordyceps pingbianensis. Table S4: Gene component of the mitogenome from Ophiocordyceps pingbianensis. Table S5: Codon usage of protein-coding genes in the mitogenome from Ophiocordyceps pingbianensis. Table S6: tRNAs in the mitogenome from Ophiocordyceps pingbianensis.
