# Supplementary material for: Mitogenomics, Phylogeny and Morphology Reveal Ophiocordyceps pingbianensis Sp. Nov., an Entomopathogenic Fungus from China
Source: Life (Basel). 2021 Jul 14;11(7):686. doi: 10.3390/life11070686 (PMC8305939; doi:10.3390/life11070686)
Supplement: Supplementary file 1 [file life-11-00686-s001.zip › Table S1.pdf]

Table S1. Specimen information and GenBank accession numbers for sequences used in this study.

Taxon	Voucher Information	Genbank Accession Number				
		nrSSU	***nrLSU***	***tef1-α***	***rpb1***	***rpb2***
Ophiocordyceps pseudocommunis	NHJ 12581	EF468973	EF468831	EF468775		EF468930
Hirsutella cryptosclerotium	ARSEF 4517	KM652066	KM652109	KM651992	KM652032	
Hirsutella fusiformis	ARSEF 5474	KM652067	KM652110	KM651993	KM652033	
Hirsutella guyana	ARSEF 878	KM652068	KM652111	KM651994	KM652035	
Hirsutella_haptospora	ARSEF 2226			KM651995	KM652036	
Hirsutella_liboensis	ARSEF 9603	KM652072	KM652115			
Hirsutella minnesotensis	3608	JPUM01000376	JPUM01000376	JPUM01000211	JPUM01000139	JPUM01000138
Hirsutella nodulosa	ARSEF 5473	KM652074	KM652117	KM652000	KM652040	
Hirsutella radiata	ARSEF 1369	KM652076	KM652119	KM652002	KM652042	
Ophiocordyceps unituberculata	YHH HU1301	KY923214	KY923212	KY923216	KY923218	
Hirsutella subulata	ARSEF 2227	KM652086	KM652130	KM652013	KM652051	
Hymenostilbe odonatae	TNS F27117		KJ878878			
Ophiocordyceps_acicularis	OSC 110988	EF468951	EF468804	EF468745	EF468853	
Ophiocordyceps agriotidis	ARSEF 5692	DQ522540	DQ518754	DQ522322	DQ522368	DQ522418
Ophiocordyceps albacongiuae	RC20	KX713633		KX713670		
Cordyceps aphodii	ARSEF 5498	DQ522541	DQ518755	DQ522323		DQ522419
Ophiocordyceps appendiculata	NBRC 106960	JN941728	JN941413	AB968577	JN992462	AB968539
Ophiocordyceps blakebarnesii	MISSOU3	KX713643	KX713608	KX713687	KX713714	
Cordyceps brunneipunctata	OSC 128576	DQ522542	DQ518756	DQ522324	DQ522369	DQ522420
Ophiocordyceps camponoti-femorati	FEMO2	KX713663	KX713590	KX713678	KX713702	
Ophiocordyceps camponoti-floridani	Flo4	KX713662	KX713591			
Ophiocordyceps camponoti-hippocrepidis	HIPPOC	KX713655	KX713597	KX713673	KX713707	
Ophiocordyceps_camponoti-nidulantis	NIDUL2	KX713640	KX713611	KX713669	KX713717	
Ophiocordyceps_camponoti-renggeri	ORENG	KX713634	KX713617	KX713671		
Ophiocordyceps_clavata	CEM1762	KJ878916	KJ878882	KJ878963	KJ878996	
Ophiocordyceps cochlidiicola	HMAS 199612	KJ878917	KJ878884	KJ878965	KJ878998	

Ophiocordyceps coenomyia	NBRC 106964	AB968385	AB968413	AB968571		AB968533
Ophiocordyceps crinalis	GDGM 17327	KF226253	KF226254	KF226256	KF226255	
Ophiocordyceps curculionum	OSC 151910	KJ878918	KJ878885		KJ878999	
Ophiocordyceps cylindrospora	MFLU 17-1961	MG553651	MG553652			MG647029
Ophiocordyceps daceti	MF01		KX713604	KX713667		
Ophiocordyceps dipterigena	OSC 151911	KJ878919	KJ878886	KJ878966	KJ879000	
Ophiocordyceps elongata	OSC 110989		EF468808	EF468748	EF468856	
Ophiocordyceps entomorrhiza	KEW 53484	EF468954	EF468809	EF468749	EF468857	EF468911
Ophiocordyceps formicarum	TNS F18565	KJ878921	KJ878888	KJ878968	KJ879002	KJ878946
Ophiocordyceps formosana	TNM F13893	KJ878908		KJ878956	KJ878988	KJ878943
Ophiocordyceps forquignonii	OSC 151902	KJ878912	KJ878876		KJ878991	KJ878945
Ophiocordyceps forquignonii	OSC 151908	KJ878922	KJ878889		KJ879003	KJ878947
Ophiocordyceps globiceps	MFLU 18-0661	MH725812	MH725830	MH727388		
Ophiocordyceps gracilis	EFCC 8572	EF468956	EF468811	EF468751	EF468859	EF468912
Ophiocordyceps gracilis	OSC 151906	KJ878923	KJ878890	KJ878969		
Ophiocordyceps heteropoda	EFCC 10125	EF468957	EF468812	EF468752	EF468860	EF468914
Ophiocordyceps iranginensis	BCC 82795			MH028186	MH028164	MH028174
Ophiocordyceps megacuculla	OSC 128578	DQ522556	DQ518770	DQ522345	DQ522391	DQ522445
Ophiocordyceps_granospora	OSC 128577	DQ522546	DQ518760	DQ522329	DQ522374	DQ522427
Ophiocordyceps kimflemingiae	SC09B	KX713631	KX713620	KX713698	KX713724	
Ophiocordyceps kniphofioides	HUA 186148	KC610790	KF658679	KC610739	KF658667	KC610717
*Ophiocordyceps konnoana_*	EFCC 7295	EF468958			EF468862	EF468915
Ophiocordyceps lanpingensis	YHOS0705	KC417458	KC417460	KC417462	KC417464	KC456333
*Ophiocordyceps lloydii_*	OSC 151913	KJ878924	KJ878891	KJ878970	KJ879004	KJ878948
Ophiocordyceps longissima	EFCC 6814		EF468817	EF468757	EF468865	
Ophiocordyceps macroacicularis	NBRC 105889	AB968390	AB968418	AB968576		AB968538
Cordyceps melolonthae	OSC 110993	DQ522548	DQ518762	DQ522331	DQ522376	
Ophiocordyceps myrmecophila	TNS 27120	KJ878929	KJ878895	KJ878975	KJ879009	
Ophiocordyceps_khaoyaiensis	HMAS 199620	KJ878927	KJ878893	KJ878973	KJ879007	

Ophiocordyceps naomipierceae	DAWKSANT	KX713664	KX713589		KX713701	
Ophiocordyceps neovolkiana	OSC 151903	KJ878930	KJ878896	KJ878976	KJ879010	
Ophiocordyceps nigrella	EFCC 9247	EF468963	EF468818	EF468758	EF468866	EF468920
Ophiocordyceps_oecophyllae	OECO1	KX713635				
Ophiocordyceps pingbianensis	**YFCC 8075**		**MT270099**	**MT270097**	**MT270098**	**MT273117**
Ophiocordyceps pruinosa	NHJ 12994	EU369106	EU369041	EU369024	EU369063	EU369084
*Ophiocordyceps pulvinata_*	TNS F 30044	GU904208		GU904209	GU904210	
Ophiocordyceps purpureostromata	TNS F18430	KJ878931	KJ878897	KJ878977	KJ879011	
*Ophiocordyceps ramosissimum_*	GZUHHN8	KJ028012		KJ028014	KJ028017	
Ophiocordyceps ravenelii	OSC 151914	KJ878932		KJ878978	KJ879012	KJ878950
Ophiocordyceps pseudorhizoidea	NHJ 12522	EF468970	EF468825	EF468764	EF468873	EF468923
Ophiocordyceps rubiginosiperitheciata	NBRC 106966	JN941704	JN941437	AB968582	JN992438	AB968544
Ophiocordyceps satoi	J7	KX713653	KX713599	KX713683	KX713711	
*Ophiocordyceps sinensis_*	EFCC 7287	EF468971	EF468827	EF468767	EF468874	EF468924
Ophiocordyceps sinensis	YN07-8	JX968027	JX968032	JX968017	JX968007	JX968012
Ophiocordyceps sobolifera	TNS F18521	KJ878933	KJ878898	KJ878979	KJ879013	
Ophiocordyceps sphecocephala	OSC 110998	DQ522551	DQ518765	DQ522336	DQ522381	DQ522432
*Ophiocordyceps sporangifera_*	MFLU 18-0658		EF468817	EF468757	EF468865	
Ophiocordyceps stylophora	OSC 110999	MH725813	MH725831	MH727389	MH727391	
Ophiocordyceps superficialis	MICH 36253	EF468982	EF468837	EF468777	EF468882	EF468931
Ophiocordyceps variabilis	ARSEF 5365	EF468983			EF468883	
Ophiocordyceps xuefengensis	GZUH 2012HN14	KC631789		KC631793	KC631798	
Ophiocordyceps yakusimensis	HMAS 199604	KJ878938	KJ878902		KJ879018	KJ878953
Ophiocordyceps_entomorrhiza	TNS 16252	KJ878941	KJ878906	KJ878986		
Paraisaria myrmicarum	IMI 393961		EU797600	EU797597		
Ophiocordyceps_citrina	TNS F18537		KJ878903	KJ878983		KJ878954
Ophiocordyceps_buquetii	HMAS 199617	KJ878940	KJ878905	KJ878985	KJ879020	
Tolypocladium inflatum	OSC 71235	EF469124	EF469077	EF469061	EF469090	EF469108
*Tolypocladium ophioglossoides_*	CBS 100239	KJ878910	KJ878874	KJ878958	KJ878990	KJ878944

Hirsutella thompsonii	ARSEF 414	KM652093	KM652139	KM652021	KM652056	
Hirsutella thompsonii	ARSEF 5412	KM652100	KM652148			
*Ophiocordyceps unilateralis*_	VIC 44303	KX713628	KX713626	KX713675	KX713730	
Hirsutella satumaensis	ARSEF 996	KM652082	KM652125	KM652008	KM652047	
Ophiocordyceps unituberculata.	YFCC HU1301	KY923214	KY923218	KY923216		KY923220
Ophiocordyceps robertsii	KEW 27083		EF468826	EF468766		
Hirsutella illustris	ARSEF 5539	KM652069	KM652112	KM651996	KM652037	
*Hirsutella lecaniicola*_	ARSEF 8888	KM652069	KM652112	KM651996	KM652037	
Hirsutella rhossiliensis	ARSEF 2931	KM652078	KM652121	KM652004	KM652043	
Hirsutella rhossiliensis	ARSEF 3747	KM652080	KM652123	KM652006	KM652045	
Hirsutella cf. haptospora	ARSEF 2228	KM652075	KM652118	KM652001	KM652041	
Hirsutella strigosa	ARSEF 2197	KM652085	KM652129	KM652012	KM652050	
Ophiocordyceps sinensis	ARSEF 6282	KM652083	KM652126	HM140637	HM140640	
