# Supplementary material for: Mitogenomics, Phylogeny and Morphology Reveal Ophiocordyceps pingbianensis Sp. Nov., an Entomopathogenic Fungus from China
Source: Life (Basel). 2021 Jul 14;11(7):686. doi: 10.3390/life11070686 (PMC8305939; doi:10.3390/life11070686)
Supplement: Supplementary file 1 [file life-11-00686-s001.zip › Table S2.pdf]

Table S2. Specimen information and GenBank accession numbers for mitogenomes used in this study.

Family	Species	Voucher Infromation	Genbank Accession Number	Mitogenome Length (bp)
Bionectriaceae	*Acremonium chrysogenum*		KF757229	27266
	Acremonium fuci	3a34	KR864757	24565
	Acremonium implicatum		KP164992	22376
	Clonostachys rosea	6792	KU668563	40921
Clavicipitaceae	*Akanthomyces lecanii*	RCEF 1005	MN904747	24643
	Epichloe festucae	AR5	KX066186	88744
	Epichloe typhina	E8	KX066185	84630
	Metacordyceps chlamydosporia	170	KF479445	25615
	Metarhizium anisopliae	ME1	AY884128	24673
	Metarhizium robertsii	ARSEF 2575	JELW01000367	24944
Cordycipitaceae	*Beauveria bassiana*	e17	KT201149	29944
	Beauveria caledonica	fhr1	KT201150	38316
	Beauveria malawiensis	k89	KT201147	44135
	Beauveria pseudobassiana	C1010	KF297618	28006
	Cordyceps bassiana		EU100742	32263
	Cordyceps brongniartii	IMBST95031	EU100743	33926
	Cordyceps cicadae	CCAD02	MH922223	56581
	Cordyceps militaris	F02	KP722505	31854
	Cordyceps tenuipes	YFCC 2017002	MK234910	31386
	Lecanicillium muscarium		AF487277	24499
	Lecanicillium saksenae		KT585676	25919
	Paecilomyces hepiali		KJ764671	24245
	Parengyodontium album	ATCC 56482	KX061492	28081
Hypocreaceae	*Hypomyces aurantius*		KU666552	71638
	Paecilomyces penicillatus	SAAS_ppe1	MK069583	27480
	Trichoderma asperellum	B05	KR952346	29999
	Trichoderma gamsii	KUC1747	KU687109	29303
	Trichoderma hamatum		MF287973	32763
	Trichoderma reesei		AF447590	42130
Lasiosphaeriaceae	*Podospora anserina*		X55026	100314
Nectriaceae	*Fusarium circinatum*		JX910419	67109
	Fusarium commune	JCM11502	LT906348	47526
	Fusarium culmorum	CBS 139512	KP827647	103844
	Fusarium fujikuroi		JX910420	46761
	Fusarium gerlachii	CBS 123666	KM486533	93428
	Fusarium graminearum		DQ364632	95676
	Fusarium mangiferae		KP742838	30629
	Fusarium oxysporum	mh2-2	MF155191	46026
	Fusarium proliferatum	ITEM2400	LT841261	46549
	Fusarium solani		JN041209	62978
	Gibberella moniliformis		JN041210	53753
	Ilyonectria destructans	2007/P/476	KU881725	42895
	Nectria cinnabarina	5175	KT731105	69895
Ophiocordycipitaceae	*Hirsutella minnesotensis*	3608	KR139916	52245
	Hirsutella rhossiliensis	USA-87-5	KU203675	62483
	Hirsutella thompsonii	ARSEF 1947	MH367296	65332
	Hirsutella vermicola	AS3.7877	KY465721	53793

	Ophiocordyceps pingbianensis	**YFCC 8075**	**MW042690**	**80359**
	Ophiocordyceps sinensis		KY622006	157539
	Tolypocladium inflatum	ARSEF 616	KY924883	24973
	Tolypocladium ophioglossoides	L2	KX455872	35159
	Tolypocladium cylindrosporum	ARSEF963	MN842262	34698
Sordariaceae	*Neurospora crassa*		KY498478	64848
