# Supplementary material for: Mitogenomics, Phylogeny and Morphology Reveal Ophiocordyceps pingbianensis Sp. Nov., an Entomopathogenic Fungus from China
Source: Life (Basel). 2021 Jul 14;11(7):686. doi: 10.3390/life11070686 (PMC8305939; doi:10.3390/life11070686)
Supplement: Supplementary file 1 [file life-11-00686-s001.zip › Table S3.pdf]

Table S3. General features in the mitogenome of *Ophiocordyceps pingbianensis.*

**Genomes Features**	**Value**
Genomes size (bp)	80,359
G + C content (%)	29.89
Numbers of protein-coding genes	15
Total length of the protein-coding genes (bp)	74081
Numbers of rRNAs	2
Sizes of rns and rnl (bp)	1652, 6444
Numbers of tRNAs	25
Lengths of tRNA genes (bp)	1811
Numbers of ORFs	24
Numbers of introns	27
Intergenic regions (bp)	21697
