# Supplementary material for: Mitogenomics, Phylogeny and Morphology Reveal Ophiocordyceps pingbianensis Sp. Nov., an Entomopathogenic Fungus from China
Source: Life (Basel). 2021 Jul 14;11(7):686. doi: 10.3390/life11070686 (PMC8305939; doi:10.3390/life11070686)
Supplement: Supplementary file 1 [file life-11-00686-s001.zip › Table S4.pdf]

Table S4. Gene component of the mitogenome from *Ophiocordyceps pingbianensis*.

Gene	Start Position	Stop Position	Length (bp)	Start Codon	Stop Codon	GC Contents (%)
trnH-GTG	445	516	70	--	--	37.5
trnM-CAT	738	809	70	--	--	37.5
nad2	961	6690	5728	ATG	TAA	31.8
orf146	6726	7166	439	AGA	TAA	28.3
orf132	7320	7715	394	TGA	TAA	31.3
nad3	7997	8410	412	ATG	TAA	21.3
atp9	8628	8852	223	ATG	TAA	32.9
orf293	9376	10257	880	AGA	TAG	24.3
orf276	10652	11482	829	ATG	TAA	26.5
cox2	9154	12319	3164	ATG	TAA	27.3
trnR-ACG	12812	12882	69	--	--	32.4
nad4L	13302	13571	268	ATG	TAA	24.4
orf306	14918	15838	919	TTT	TAG	26.4
orf412	16154	17392	1237	CAA	TAA	25.7
nad5	13571	18547	4975	ATG	TAG	26.3
orf371	19838	20953	1114	ATG	TAA	30.2
orf508	22541	24067	1525	ATG	TAG	21.9
orf310	25908	26840	931	TAC	TAA	25.7
orf473	27376	28797	1420	AAA	TAA	24.0
cob	25515	30126	4610	ATG	TAA	26.7
trnC-GCA	30273	30344	70	--	--	31.9
orf341	32340	33365	1024	AGA	TAA	24.3
orf394_2	33838	35022	1183	AAA	TAA	29.3
orf332	35279	36277	997	AAA	TAA	22.8
cox1	31321	36717	5395	ATG	GCT	29.0
orf105	37420	37737	316	ATG	TAG	43.1
orf114	37910	38254	343	ATG	TAA	38.6
orf623	38637	40508	1870	ATG	TAA	28.0
orf394	40963	42147	1183	ATG	TAA	24.0
cox1	42215	42682	466	CAC	TAG	31.2
trnR-TCT	43016	43086	69	--	--	31.0
nad1	43810	46106	2295	ATG	TAA	25.8
orf164	47506	48000	493	AAC	TAA	38.6
orf108	48347	48673	325	ATG	TAA	38.2
nad4	46888	50054	3165	ATG	TAG	28.4
orf374	50024	51148	1123	ATG	TAG	32.4
atp8	51985	52131	145	ATG	TAA	19.7
orf358	52651	53727	1075	AAC	TAG	27.9
atp6	52309	54476	2166	ATG	TAA	27.6
rns	55382	57035	1652	--	--	33.4
trnY-GTA	57072	57155	82	--	--	38.1
trnD-GTC	57532	57604	71	--	--	37.0
trnS-GCT	58140	58220	79	--	--	37.0
trnN-GTT	58583	58654	70	--	--	36.1
orf302	59025	59933	907	AAA	TAG	23.5
orf757	60749	63022	2272	GTG	TAG	35.5
cox3	58806	63672	4865	ATG	TAA	32.3
trnG-TCC	64294	64364	69	--	--	36.6
nad6	64741	65541	799	TTG	TAG	25.3

trnV-TAC	65747	65818	70	--	--	29.2
trnI-GAT	66572	66643	70	--	--	31.9
trnS-TGA	66970	67055	84	--	--	37.2
trnW-TCA	67168	67239	70	--	--	29.2
orf268	67623	68429	805	ATG	TAA	24.5
trnP-TGG	68852	68924	71	--	--	50.7
rps3	72562	74457	1894	ATG	TAG	24.2
rnl	69035	75480	6444	--	--	28.8
trnT-TGT	75484	75554	69	--	--	38.0
trnE-TTC	75964	76036	71	--	--	45.2
trnM-CAT	76040	76111	70	--	--	37.5
trnM-CAT	76113	76185	71	--	--	35.6
trnL-TAA	76224	76306	81	--	--	44.6
trnF-GAA	77347	77418	70	--	--	31.9
trnK-TTT	78065	78137	71	--	--	35.6
trnA-TGC	78744	78815	70	--	--	36.1
trnL-TAG	79115	79198	82	--	--	35.7
trnQ-TTG	79862	79935	72	--	--	29.7
