# Supplementary material for: Mitogenomics, Phylogeny and Morphology Reveal Ophiocordyceps pingbianensis Sp. Nov., an Entomopathogenic Fungus from China
Source: Life (Basel). 2021 Jul 14;11(7):686. doi: 10.3390/life11070686 (PMC8305939; doi:10.3390/life11070686)
Supplement: Supplementary file 1 [file life-11-00686-s001.zip › Table S5.pdf]

Table S5. Codon usage of protein-coding genes in the mitogenome from *Ophiocordyceps pingbianensis*.

AmAcid	Codon	Frequence	RSCU
Leu	UUA	991	3.88
Arg	AGA	290	3.6
Pro	CCU	223	2.37
Ala	GCU	328	1.99
Ser	UCU	346	1.94
Gly	GGU	324	1.93
Val	GUA	294	1.91
Thr	ACU	287	1.87
Ser	AGU	328	1.84
Asp	GAU	405	1.75
Tyr	UAU	587	1.72
Thr	ACA	262	1.71
Ile	AUA	787	1.7
Asn	AAU	758	1.68
Lys	AAA	890	1.68
Phe	UUU	703	1.66
His	CAU	218	1.62
Gln	CAA	272	1.62
Trp	UGA	156	1.62
Glu	GAA	395	1.61
Val	GUU	234	1.52
Cys	UGU	133	1.43
Gly	GGA	234	1.39
Ala	GCA	211	1.28
Ser	UCA	222	1.24
Ile	AUU	518	1.12
Pro	CCA	98	1.04
Met	AUG	269	1
Arg	CGU	74	0.92
Leu	CUU	197	0.77
Leu	CUA	149	0.58
Cys	UGC	53	0.57
Ser	AGC	96	0.54
Leu	UUG	119	0.47
Arg	CGA	38	0.47
Arg	AGG	38	0.47
Ala	GCC	66	0.4
Arg	CGC	32	0.4
Glu	GAG	96	0.39
His	CAC	51	0.38
Gln	CAG	64	0.38
Trp	UGG	37	0.38
Gly	GGG	60	0.36
Phe	UUC	145	0.34
Val	GUG	52	0.34
Ala	GCG	54	0.33
Asn	AAC	147	0.32
Lys	AAG	168	0.32
Gly	GGC	54	0.32
Pro	CCG	29	0.31

Pro	CCC	26	0.28
Tyr	UAC	95	0.28
Thr	ACG	39	0.25
Asp	GAC	59	0.25
Val	GUC	35	0.23
Ser	UCG	41	0.23
Ser	UCC	39	0.22
Leu	CUG	49	0.19
Ile	AUC	82	0.18
Thr	ACC	26	0.17
Arg	CGG	12	0.15
Leu	CUC	29	0.11
