# Supplementary material for: Mitogenomics, Phylogeny and Morphology Reveal Ophiocordyceps pingbianensis Sp. Nov., an Entomopathogenic Fungus from China
Source: Life (Basel). 2021 Jul 14;11(7):686. doi: 10.3390/life11070686 (PMC8305939; doi:10.3390/life11070686)
Supplement: Supplementary file 1 [file life-11-00686-s001.zip › Table S6.pdf]

Table S6. tRNAs in the mitogenome from *Ophiocordyceps pingbianensis.*

tRNA Type	Anticodon	Numbers
His	GTG	1
Met	CAT	3
Arg	ACG	1
Cys	GCA	1
Arg	TCT	1
Tyr	GTA	1
Asp	GTC	1
Ser	GCT	1
Asn	GTT	1
Gly	TCC	1
Val	TAC	1
Ile	GAT	1
Ser	TGA	1
Trp	TCA	1
Pro	TGG	1
Thr	TGT	1
Glu	TTC	1
Leu	TAA	1
Phe	GAA	1
Lys	TTT	1
Ala	TGC	1
Leu	TAG	1
Gln	TTG	1
